# CatWalk gait analysis in a rat model of multiple sclerosis

**DOI:** 10.1186/s12868-016-0317-0

**Published:** 2016-11-30

**Authors:** Sabine Herold, Prateek Kumar, Klaus Jung, Irina Graf, Henrike Menkhoff, Xenia Schulz, Mathias Bähr, Katharina Hein

**Affiliations:** 1 Department of Neurology, University Medicine Göttingen, Robert-Koch Straße 40, 37073 Göttingen, Germany; 2 Department of Medical Biometry and Statistical Bioinformatics, University Medicine Göttingen, Robert-Koch Straße 40, 37073 Göttingen, Germany; 3 Institute of Animal Breeding and Genetics, University of Veterinary Medicine Hannover, Hannover, Germany; 4 Department of Neurology, University Hospital, Robert-Koch-Strasse 40, 37075 Göttingen, Germany

**Keywords:** Multiple sclerosis, Experimental autoimmune encephalomyelitis, CatWalk, Cortison, Spinal cord, Behavioral test

## Abstract

**Background:**

Myelin oligodendrocyte glycoprotein (MOG)-induced experimental autoimmune encephalomyelitis (EAE) is a widely used animal model for multiple sclerosis. The characteristic feature of the MOG-EAE model in Brown Norway rats is consistent involvement of the spinal cord resulting in limb paresis. The aim of the study was to investigate whether early subclinical gait abnormalities are present in this animal model and can be detected by CatWalk XT, a fully automated gait analysis system. Furthermore, we investigated the usability of CatWalk system for treatment studies.

**Results:**

Our gait analysis showed no preclinical abnormalities in MOG-EAE animals. Nevertheless, we characterized a combination of gait parameters that display a high predictive capacity in regard to disease onset. Our detailed histopathological analysis of the spinal cord revealed that lesion formation starts in the lumbar region and propagates toward the cervical part of the spinal cord during the disease course. In the treatment study, the stabilization of gait parameters under the treatment with methylprednisolone was detected in CatWalk as well as in traditional EAE-scoring system.

**Conclusions:**

The results from CatWalk test indicate no benefit of lab-intensive automated gait system in EAE-model with chronic-progressive disease course as well as in therapeutic studies with pronounced effect on the severity of clinical symptoms. However, due to its quantitative and objective nature this system may display a refined test to detect small but functional relevant changes in regeneration-orientated studies.

## Background

Multiple sclerosis (MS) is an autoimmune inflammatory demyelinating disease of the central nervous system (CNS). Clinical symptoms of the disease are quite heterogeneous. In MS-patients, the expanded disability status scale (EDSS) is used to monitor CNS-related dysfunctions [1]. The score is calculated by testing different functional systems of the CNS and thereby reflects an overall estimation of patient’s disability. Current gait analysis in MS-patients with detailed investigation paradigms revealed that abnormalities in gait occur far earlier during the disease course than expected and that these subclinical abnormalities are neglected by the EDSS scoring paradigm, especially in the lower range of the scale [2, 3]. A scoring system similar to EDSS is used for clinical evaluation of the CNS-damage in animal models of MS [4]. The most commonly used animal model to mimic the human disease is experimental autoimmune encephalomyelitis (EAE) [5, 6]. In the present experiments we employed an EAE model in Brown Norway (BN) rats. Active immunization of BN-rats with myelin oligodendrocyte glycoprotein (MOG) leads to the development of clinical symptoms within 2 weeks after immunization. This animal model displays many of the pathological aspects seen in MS-patients [7], with inflammation, demyelination and neuronal damage being restricted to the spinal cord and optic nerve. The traditional scoring system in EAE ranges from 0 to 4 depends on the severity of limbs paresis and therefore reflects spinal cord involvement. Our previous studies on the visual pathway in this animal model suggest that treatment with neuroprotective substances should be started early even in the pre-clinical stage of the disease [8–10]. Thus, in the present project we aimed to evaluate early clinically not detectable abnormalities in locomotion using automated gait analysis in MOG-immunized animals in order to identify a specific time point for the start of neuroprotective therapies. To that end, we established the CatWalk fully automated gait analysis system (CatWalk XT, Noldus Information Technology, Netherlands) as a quantitative test in MOG-EAE animal model. Furthermore, we investigated the timeline of histopathological changes in the spinal cord in preclinical as well as in clinical stage of the disease. Moreover, to investigate the usability of Catwalk to detect improvement of locomotion abnormalities we applied this automated behavioral test in the treatment study with methylprednisolone (Mps), a standard therapy for MS-relapses.

## Methods

### Animals

Forty-two female BN rats in the age of 8–10 weeks were used for all experiments. Animals were obtained from Charles River (Sulzfeld, Germany) and kept under environmentally controlled conditions. Food and drinking water was given with free access. All animal experiments were controlled by and performed in accordance to the local authorities for animal research LAVES (*Niedersächsisches Landesamt für Verbraucherschutz und Lebensmittelsicherheit, Reg. nr. G13/1332*), Oldenburg, Germany.

### Immunization, evaluation and treatment of animals

Animals were anesthetized by inhalation of isofluorane (Abbott, Wiesbaden; Germany) and injected intradermally at the base of the tail with 200 µl inoculum containing 50 µg recombinant rat MOG (kindly provided by C. Stadelmann, Department of Neuropathology, University Medicine Göttingen, Germany) in saline emulsified with complete Freund’s adjuvant (Sigma-Aldrich, St. Louis; MO) containing 200 µg heat-inactivated *Mycobacterium tuberculosis* (strain H37 RA, Difico Laboratories, Detroit, MI). Sham-immunized animals received incomplete Freund’s adjuvant without MOG. Animals were scored for clinical signs of EAE and weighed daily. EAE-symptoms were scored as follows: *0 no symptoms, 0.5 distal tail paresis, 1.0 complete tail paralysis, 1.5 complete tail paralysis and partial paresis of hindlimbs, 2.0 complete hindlimb paresis on one side, 2.5 complete paresis of hindlimbs, 3.0 complete paresis of both hindlimbs 3.5 complete paresis of hindlimbs and partial paresis of forelimbs, 4.0 tetraparesis or death*.

In the treatment study, 18 MOG-immunized rats were randomly assigned into two different treatment groups (9 animals per group). Animals were treated with intraperitoneal (i.p.) injections of Mps (20 mg/kg; Urbason®, Hoechst Marion Roussel, Frankfurt/Main, Germany) or vehicle (0.9% NaCl) on days 1–3 of the disease. To exclude interference with the immunization procedure two sham-immunized animals were run in parallel.

### Locomotion-data acquisition and analysis

**Fig. 1:**
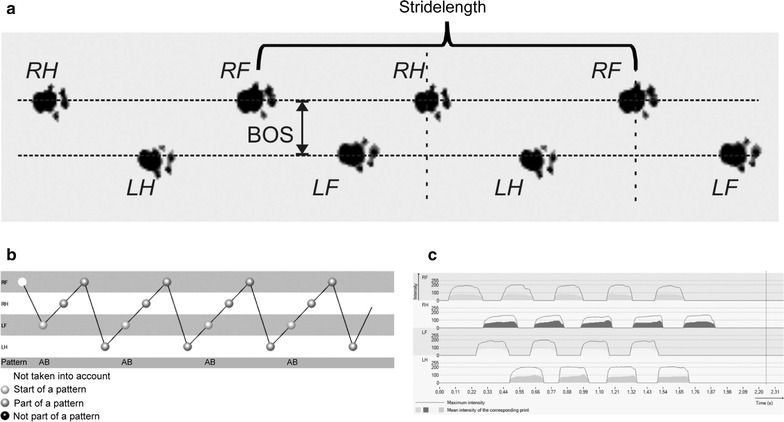
Dynamic parameters for gait analysis in BN-rats. **a** Section from original footprints recorded from a healthy BN-rat. The animal is running to the right. **b** Normal step-pattern in healthy BN-rats is quite constant and usually gives a regularity index of almost 100%. **c** Mean footprint intensity from all four limbs is comparable under healthy conditions. *RF* right forelimb, *RH* right hindlimb, *LF* left forelimb, *LH* left hindlimb, *BOS* base of support

Twenty-four animals were trained prior to recording for at least 1 week, meaning that the animals were habituated to the behavioral testing room and each animal performed at least 3 runs per day. Training was conducted by the same person who performed the final experiment and tested the animals. Before recording, animals were allowed to habituate and cross the walkway 1–2 times. Quantitative fully automated gait recording with the CatWalk System (CatWalk XT, Noldus Information Technology, Netherlands) was performed 1 h before immunization and continued from day 4 post immunization (4 dpi) onwards until day 12 or 16 dpi depending on the severity of EAE-symptoms. Animals had to pass the walkway until complete hindlimb paresis was developed (corresponding to the clinical score of 3). In the treatment study animals were sacrificed on day 5 of EAE or reaching complete paresis with inability to perform successful runs in the CatWalk. Therefore, sample size per time point differs. Number of animals that successfully performed in the CatWalk was: 0 dpi = 24, 4 dpi = 18, 6 dpi = 15, 8 dpi = 9, 9 dpi = 18, 10 dpi = 15, 11 dpi = 11, 12 dpi = 8, 12 dpi = 2, 14 and 16 dpi = 1. Time of clinical manifestation differs between animals. The Noldus CatWalk system has been described in detail elsewhere [11]. In brief, the CatWalk System consists of an enclosed walkway (glass plate) that is illuminated by fluorescent light. The system is furthermore equipped with a high speed color camera connected to a computer with the appropriate detection software (CatwalkXT9.1). Animals were individually placed into the walkway and each animal was allowed to move freely in both directions. For the detection of all parameters used in the experiments the camera gain was set to 20 and the detection threshold to 0.1. All runs with a run duration between 0.50 and 5.00 s for complete walkway and a maximum allowed speed variation of 60% were considered as successful runs. For each animal and analyzed time point 3 compliant runs were acquired per trial. Compliant runs were classified for all limbs and statistically analyzed. The software is able to detect several dynamic parameters of an animal’s walk. In the present study parameters that are linked to coordination and balance were predominantly analyzed (Fig. 1). Paw statistics were not included into the analysis. Stride length gives the distance between one limb touching the glass blade in one step cycle and touching the plate again in the next. Base of support is the distance of both forelimbs or of both hindlimbs to each other indicating the inclination of legs. The footprint pattern illustrates the composition of one step cycle. Under normal conditions the used pattern in rodents can differ because of disease or stress conditions [11]. The pressure which is given to each paw is calculated by foot print intensity and partially by the area which is covered by the footprint. These parameters and further dynamic parameters like run duration or swing speed were statistically analyzed. All these parameters were statistically quantified for all time points investigated.

### Histopathology and Immunohistochemistry

Three individual animals were sacrificed for each time point. Histopathological evaluation of spinal cord was performed on paraformaldehyde-fixed paraffin-embedded 2 µm sections. Spinal cord was divided in a cervical, thoracic and lumbar region. Subsequently, we subdivided the cervical part in 3 tissue blocks, the thoracic region in 6–8 small tissue blocks and the lumbar part in 3–4 tissue blocks. The number of tissue blocks from each anatomical region varies. Finally, cervical, thoracic and lumbar tissue blocks were embedded in a standardized sequence in one paraffin block each. Thus, this technique enables us to evaluate a greater surface area of anatomical regions. Luxol Fast Blue staining was performed to assess demyelination. Staining procedure is described in detail elsewhere [9]. Immunohistochemistry was performed on adjacent paraffin sections. ED-1 positive macrophages/activated microglia (MCA341R, Biorad AbD Serotec GmbH, Puchheim, Germany; diluted 1:500), CD-3 positive T-lymphocytes (BZL03543, Biozol Diagnostica GmbH, Eching, Germany; diluted 1:100) and β-amyloid precursor protein (APP) positive axons (MAB348, Chemicon, Ford, UK; diluted 1:1000) as a marker for acute axonal damage were stained in all tissue sections. Anti-rabbit-biotinylated antibody was used as a secondary antibody. Spleen sections served as positive control for ED1 staining.

### Microscopic techniques and evaluation of staining

Images of spinal cord sections were taken with Olympus dot Slide 2.1 system, based on the upright BX51 microscope and a high resolution color camera (Olympus Deutschland GmbH, Hamburg, Germany). Mosaic-Images of complete spinal cord cross sections were taken with 20× objective. For each anatomic position within the optic nerve or spinal cord at least 3 individual sections were imaged.

Evaluation of demyelination was measured as previously described [7, 12]. The evaluation of ED1 positive cells was performed using the following score: (0) no labelled cells; (1) a few ED1 positive cells (number countable) in at least one of the three cross sections of the spinal cord; (2) infiltration of less than 10% of the ON cross section area with ED1 positive cells (number not countable) in at least one of the three cross sections of the spinal cord; (3) infiltration of 10–50% of the spinal cord cross section area with labelled ED1 positive cells in at least one of the three cross sections of the spinal cord; (4) infiltration of 80% of the cross section area; (5) infiltration of more than 80% of the cross section area in at least one of the three cross sections of the spinal cord. The number of CD3-positive cells and β-APP positive axons was counted in three different spinal cord sections per region.

## Statistical methods

The distribution of each study parameter was described by its mean ± standard deviation, separately for each time after immunization. The mean time courses (±standard deviation) were plotted for each parameter. The effect of time and group as well as their interaction was assessed by analysis of variance for longitudinal data, i.e. by repeated measures ANOVA. Group effects were further studied at each time by means of *t* tests. Beforehand, the normality of each parameter was checked by normal quantile–quantile plots. The significance level for the analysis of variance models was set to α = 5%. In the case of a significant global group effect, *t* tests were also performed at a significance level of 5%, and at Bonferroni–Holm adjusted levels otherwise. The effect of elapsed time and all other study parameters of the disease status of the animals were assessed by univariate and multivariate logistic regression for clustered data to take the repeated measures situation into account.

All analyses were performed using the statistic software R (www.r-project.org), the analysis of variance and logistic regression was modelled by means of generalized estimating equation using the R-package ‘geepack’.

The datasets supporting the conclusions of this article are included within the article.

## Results

### Assessment of locomotion in preclinical and clinical EAE

**Fig. 2:**
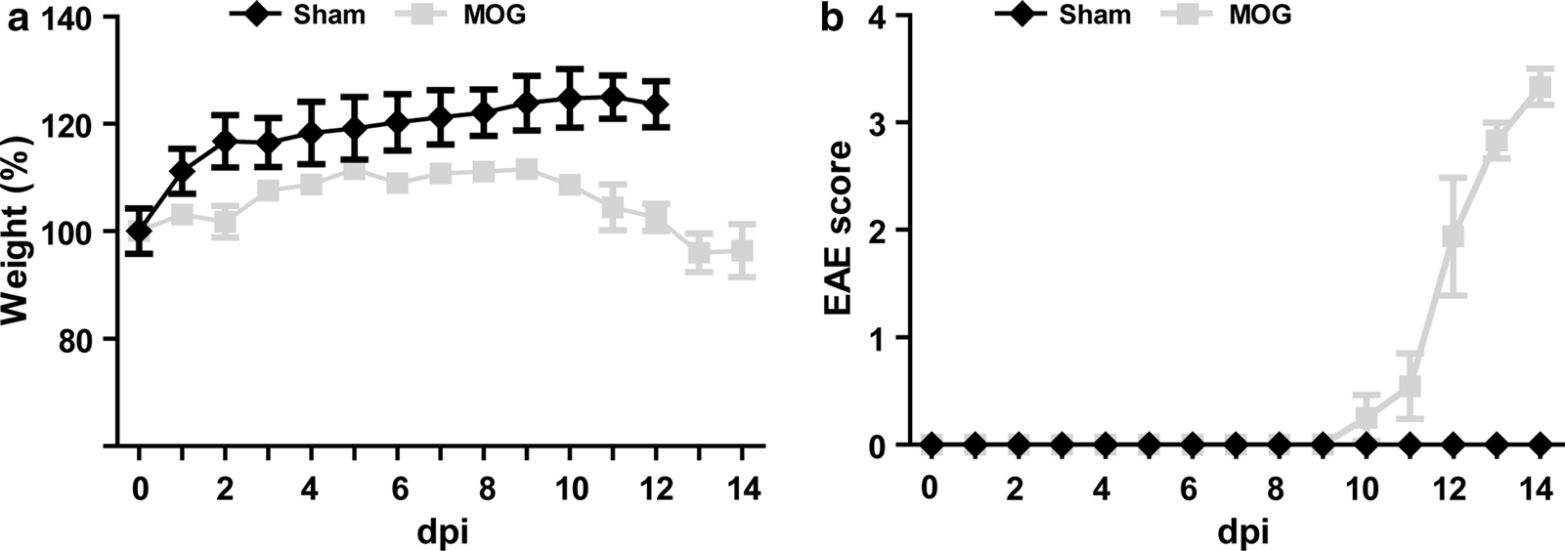
Weight (**a**) and EAE-score (**b**) curves of sham- and MOG-immunized animals. Sham-immunized healthy animals gained weight during the experiment and did not develop clinical signs of EAE. MOG-immunized animals slightly lost weight at EAE onset

After immunization, animals were scored for clinical signs of EAE and weighed daily. As expected sham-immunized animals gained weight and did not display any disabilities during observation period whereas MOG-immunized animals lost weight at the time of clinical manifestation of EAE (Fig. 2). The time of disease onset in MOG-immunized animals was at day 11.3 ± 0.82 post immunization in the first part of the experiment. Because weight loss did not excess more than 10% of total body weight in any animal this phenomenon does not interfere with gait recording.

As mentioned above the aim of the study was to quantify early, in regard to the EAE scoring paradigm subclinical abnormalities in the gait of MOG-EAE animals compared to sham-immunized control animals. Therefore, we quantified statistically a great cohort of run-parameters in sham- and MOG-immunized animals.

**Fig. 3:**
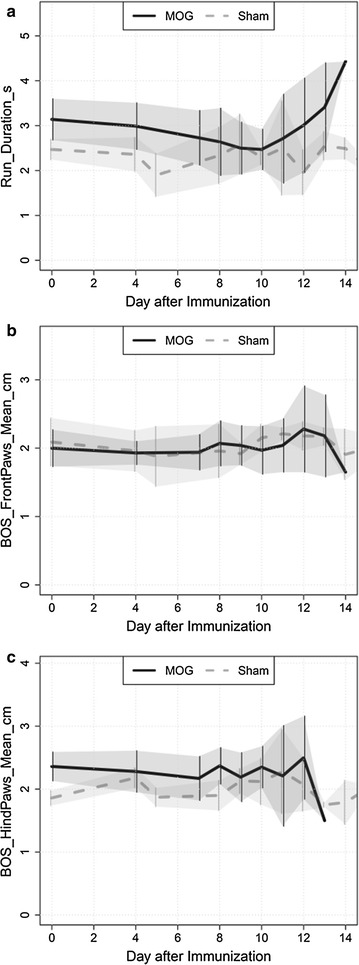
Comparison of run duration and inclination of legs. **a** run duration time is overall higher in the MOG-immunized than in the control group. Base of support (BOS) of forelimbs (**b**) as well as BOS of hindlimbs (**c**) were unchanged in preclinical and clinical EAE compared to control animals

A significant group-effect (P < 0.001) was detected for the parameter run duration whereas neither time nor interaction of time and group revealed significant impact (*P* = 0.069 for time and P = 0.349 for group-by-time interaction) (Fig. 3). Analysis of base of support (BOS) of fore- and hind-limbs did not reveal any significant differences in time (P = 0.241 for forelimbs and P = 0.473 for hindlimbs) or group (P = 0.795 for forelimbs and P = 0.051 for hindlimbs) or the interaction of time and group (P = 0.444 for forelimbs and P = 0.491 for hindlimbs; Fig. 3).

**Fig. 4:**
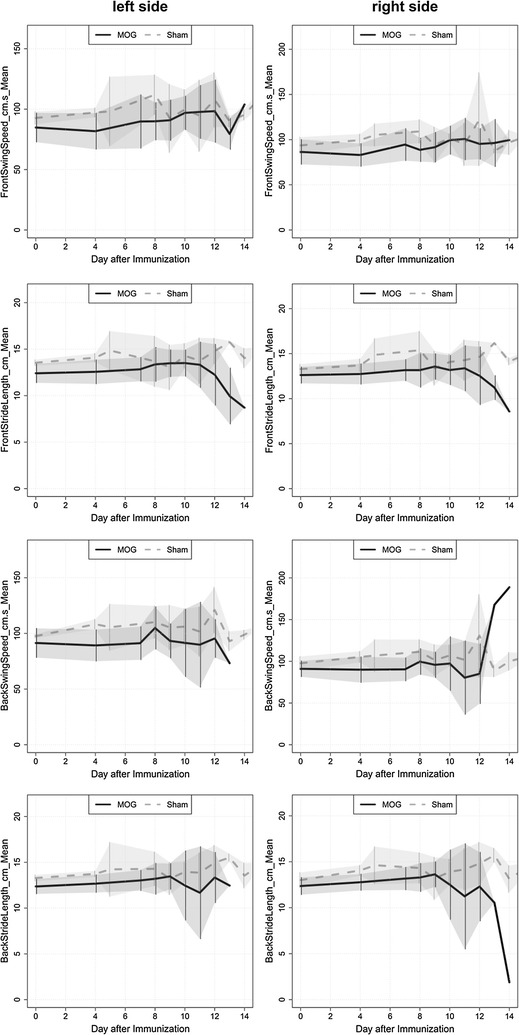
Time-specific comparison of dynamic gait parameter in MOG-versus sham-immunized animals

In addition, the straight length of hindlimbs and forelimbs as well as the swing speed of fore- and hind-limbs were quantified. Based on EAE-scoring paradigm these gait-parameters appeared to be highly affected at clinical manifestation of EAE. A significant difference between MOG and sham animals was detected for stride length and swing speed at time point 4 for the left forelimbs as well as hindlimbs (Table 1). This is due to overall higher values in the control group compared to the MOG group (Fig. 4).

**Table 1:** Table representing the significantly different parameters between MOG-immunized and control group at the specific time point

Side	Parameter	Time	Control	Treatment	P	p.Holm
Left	BackMaxContactArea_cm2_Mean	9	0.98 ± 0.07	0.72 ± 0.17	0.0024	0.0144
Left	BackMaxIntensity_Mean	9	193.69 ± 8.71	166.56 ± 22.74	0.0062	0.0372
Left	BackPrintArea_cm2_Mean	8	1.67 ± 0.14	1.11 ± 0.37	0.0038	0.0204
Left	BackPrintArea_cm2_Mean	9	1.35 ± 0.11	0.96 ± 0.28	0.0034	0.0204
Left	BackStrideLength_cm_Mean	4	13.74 ± 0.32	12.67 ± 0.97	0.0035	0.0245
Left	BackSwing_s_Mean	4	0.13 ± 0	0.15 ± 0.02	0.001	0.006
Left	BackSwingSpeed_cm.s_Mean	4	108.29 ± 4.51	89.21 ± 14.17	9e−04	0.0063
Left	FrontMaxContactArea_cm2_Mean	10	1.44 ± 0.1	1.08 ± 0.22	0.004	0.024
Left	FrontMaxIntensity_Mean	4	200.38 ± 3.35	186.52 ± 20.48	0.0097	0.046
Left	FrontMaxIntensity_Mean	8	195.44 ± 10.15	163.39 ± 22.45	0.0092	0.046
Left	FrontMaxIntensity_Mean	10	203.06 ± 2.04	172.51 ± 24.26	4e−04	0.0024
Left	FrontMaxIntensity_Mean	12	204.88 ± 7.67	169.93 ± 28.29	0.0112	0.046
Left	FrontPrintArea_cm2_Mean	10	1.66 ± 0.12	1.28 ± 0.19	0.008	0.04
Left	FrontPrintArea_cm2_Mean	11	1.94 ± 0.16	1.42 ± 0.4	0.0066	0.0396
Left	FrontStrideLength_cm_Mean	4	14.08 ± 0.47	12.56 ± 1.3	0.0047	0.0376
Left	FrontSwingSpeed_cm.s_Mean	4	97.23 ± 3.82	81.76 ± 14.98	0.0016	0.0128
Right	BackMaxContactArea_cm2_Mean	8	1.29 ± 0.11	0.78 ± 0.28	0.0012	0.0072
Right	BackMaxContactArea_cm2_Mean	9	0.95 ± 0.05	0.72 ± 0.29	0.0071	0.0284
Right	BackMaxContactArea_cm2_Mean	11	1.39 ± 0.18	0.74 ± 0.35	0.0033	0.0165
Right	BackPrintArea_cm2_Mean	8	1.72 ± 0.12	1.06 ± 0.38	0.001	0.005
Right	BackPrintArea_cm2_Mean	9	1.32 ± 0.08	0.97 ± 0.41	0.0041	0.0164
Right	BackPrintArea_cm2_Mean	11	1.95 ± 0.13	1.02 ± 0.47	2e−04	0.0012
Right	FrontMaxContactArea_cm2_Mean	8	1.63 ± 0.04	0.94 ± 0.32	2e−04	0.0012
Right	FrontMaxContactArea_cm2_Mean	9	1.45 ± 0.11	1.12 ± 0.28	0.0085	0.034
Right	FrontMaxContactArea_cm2_Mean	11	1.78 ± 0.1	1.23 ± 0.4	0.0012	0.006
Right	FrontMaxIntensity_Mean	8	205.75 ± 1.47	152.01 ± 36.72	0.0023	0.0138
Right	FrontMaxIntensity_Mean	10	197.77 ± 8.23	167.24 ± 30.55	0.0064	0.032
Right	FrontMaxIntensity_Mean	11	206.04 ± 1.86	173.16 ± 35.76	0.0123	0.0492
Right	FrontPrintArea_cm2_Mean	8	1.84 ± 0.08	1.15 ± 0.33	2e−04	0.0012
Right	FrontPrintArea_cm2_Mean	9	1.63 ± 0.09	1.31 ± 0.29	0.0041	0.0164
Right	FrontPrintArea_cm2_Mean	11	2.08 ± 0.15	1.45 ± 0.39	0.0019	0.0095
Right	FrontSwing_s_Mean	4	0.14 ± 0.01	0.17 ± 0.03	0.0082	0.0492
Right	StepSequence_RegularityIndex_percent	4	99.47 ± 0.92	95.94 ± 3.9	0.003	0.018

Besides these parameters we quantified: print area, maximal intensity of footprints, maximal contact area, step sequence number of patterns and step sequence Regularity index of all four limbs.

### Predictive power of gait-analysis during disease induction is restricted to the right-sided extremities

**Fig. 5:**
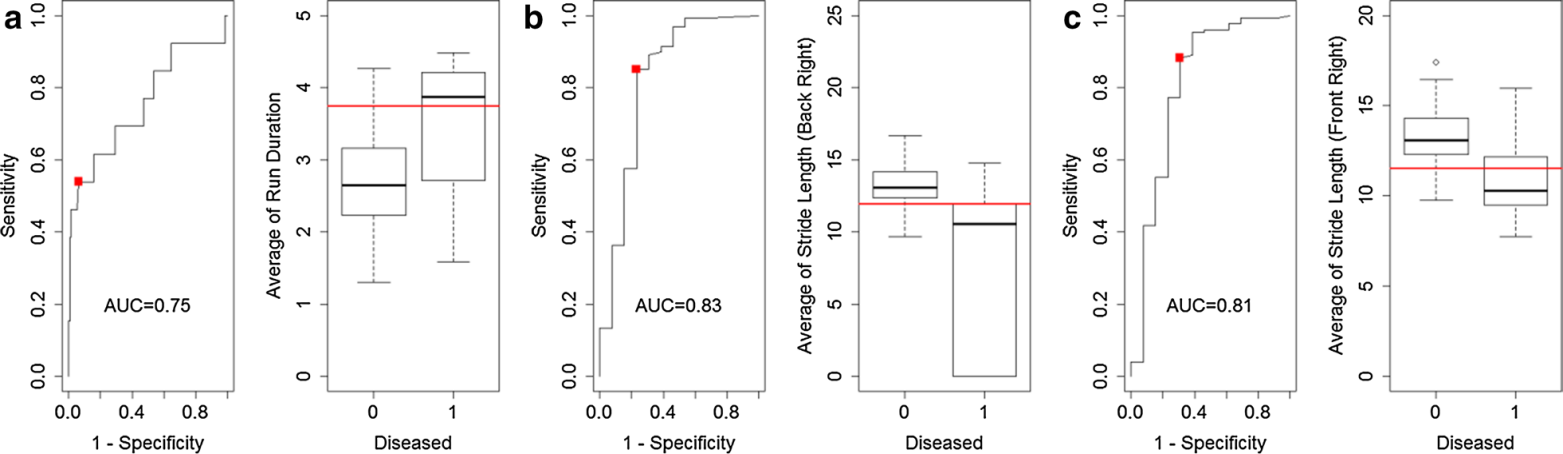
ROC curves of parameters in the MOG-EAE group

Logistic regression yielded that the strongest predictive power with regard to the disease status of the animals was given by the parameters run duration, average of stride length (front right) and the average of stride length (back right). Their predictive power was also demonstrated in ROC curve analyses yielding area under the curve (AUC) values between 0.75 (run duration), 0.81 (average of stride length, front right) and 0.83 (average of stride length, back right) (Fig. 5).

**Fig. 6:**
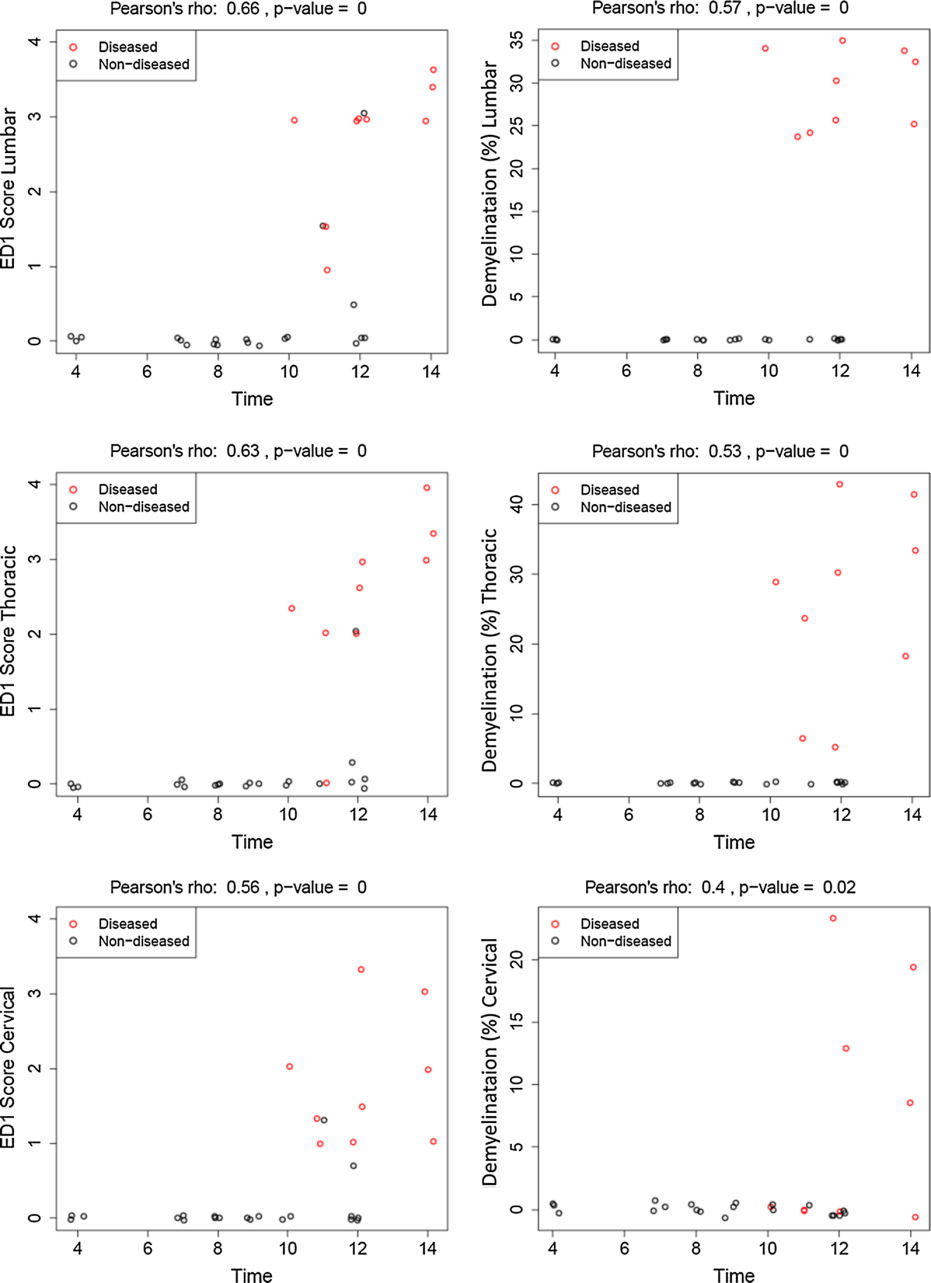
Histopathology of the spinal cord of sham-immunized and MOG-immunized animals. Inflammation (ED1) as well as demyelination start in the lumbar part of the spinal cord and propagates toward the cervical area. There are no histopathological changes detectable in the preclinical phase of EAE. *Circles* represent values for one individual animal

Taking together the data validated that there is no single gait-parameter which can be used as a predictive marker. Although all of the gait parameters shown above have a high predictive power, they are only useful if they are analyzed together to gain a reliable tool for prediction of disease onset.

### Histopathological changes in the spinal cord

**Fig. 7:**
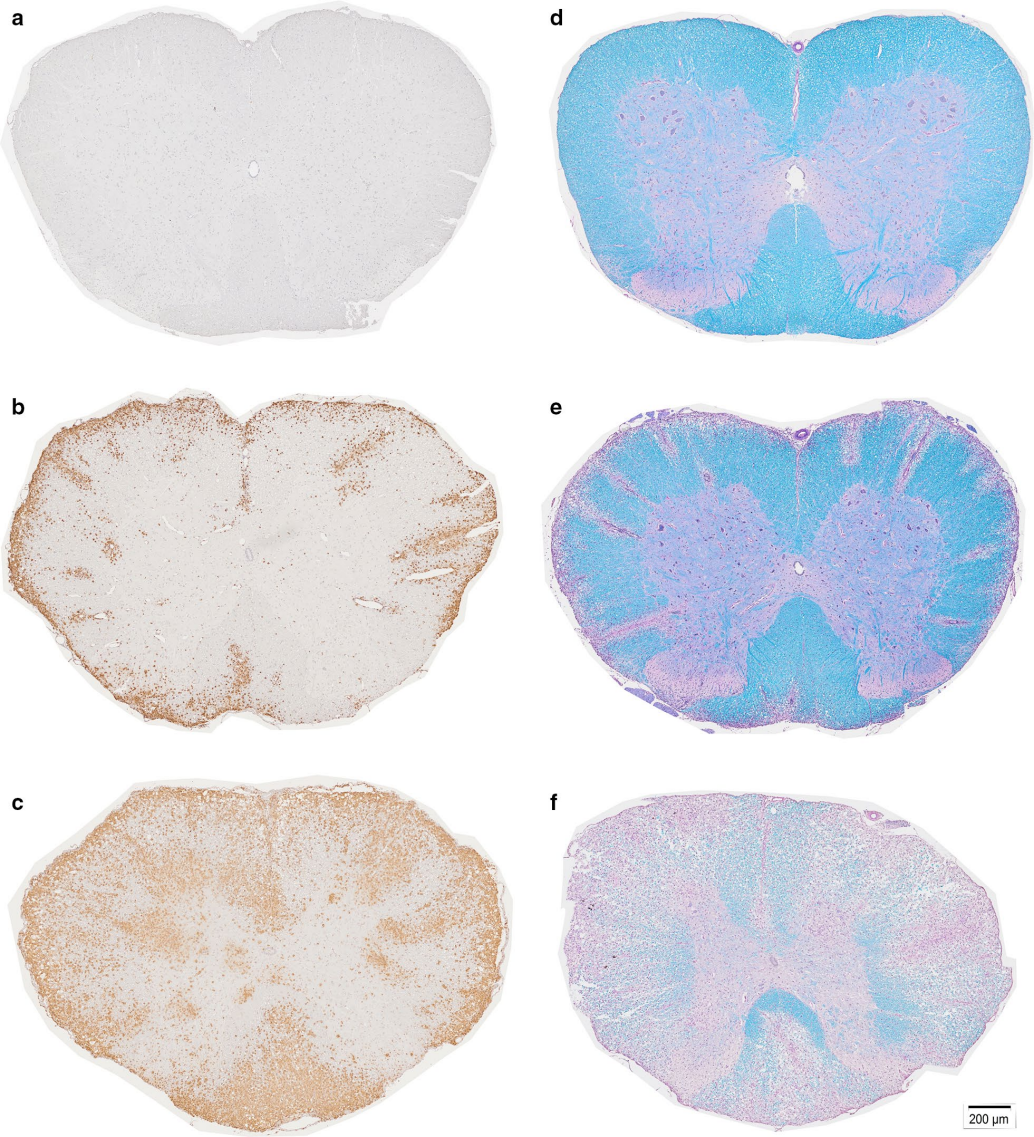
Spinal cord (SC) histopathology. Representative examples of ED1-staining in animal at 4 days post immunization (dpi) shows no abnormalities (**a**); small level of inflammatory infiltration at 11d pi (**b**); in contrast heavily infiltrated SC section at 14 dpi (**c**). Representative Luxol Fast Blue-stained cross-section of the SC shows intact myelin (*blue*) in animal at 4 dpi (**d**) only small areas of demyelination, with mainly intact myelin (*blue*) on 11 dpi (**e**); a cross-section from diseased animal on day 14 dpi (**f**) shows extensively demyelinated areas (*purple*)

For each time point three animals were sacrificed and the spinal cord was taken out for histopathological analysis. No obvious histopathological changes in the spinal cord were observed before clinical manifestation of the disease. The inflammatory lesions were characterized by an infiltration of macrophages. Quantification of CD3-positive cells revealed that only a minority of infiltrating cells were CD3 + T-lymphocytes (13.6 ± 4.72; 56.33 ± 11.02; 105.5 ± 16.21 cells per section at day 11 dpi, 12 dpi and 14 dpi respectively). Within the lesions, myelin was lost and the extent of inflammation was consistent with the degree of inflammation (Fig. 7). Demyelinating lesions were typically located around submeningeal veins and relatively large vessels rather than in parenchymal vessels and underlying grey matter. Immunohistochemistry for beta APP indicated acute axonal damage starts at disease onset (116.44 ± 70.64/section for 11 dpi) and increased during the disease course (298.66 ± 186.71/section for 12 dpi and 304.56 ± 41.44/section 14 dpi respectively). Analysis of the different regions of the spinal cord revealed that lesion formation starts in the lumbar region of the spinal cord at the day of clinical manifestation of EAE and propagate toward the cervical portion of the spinal cord during the disease course (Fig. 6). Before the onset of first clinical symptoms we found no histopathological abnormalities in the spinal cords for all time points analyzed.

### Evaluation of clinical parameters during the steroid treatment

**Fig. 8:**
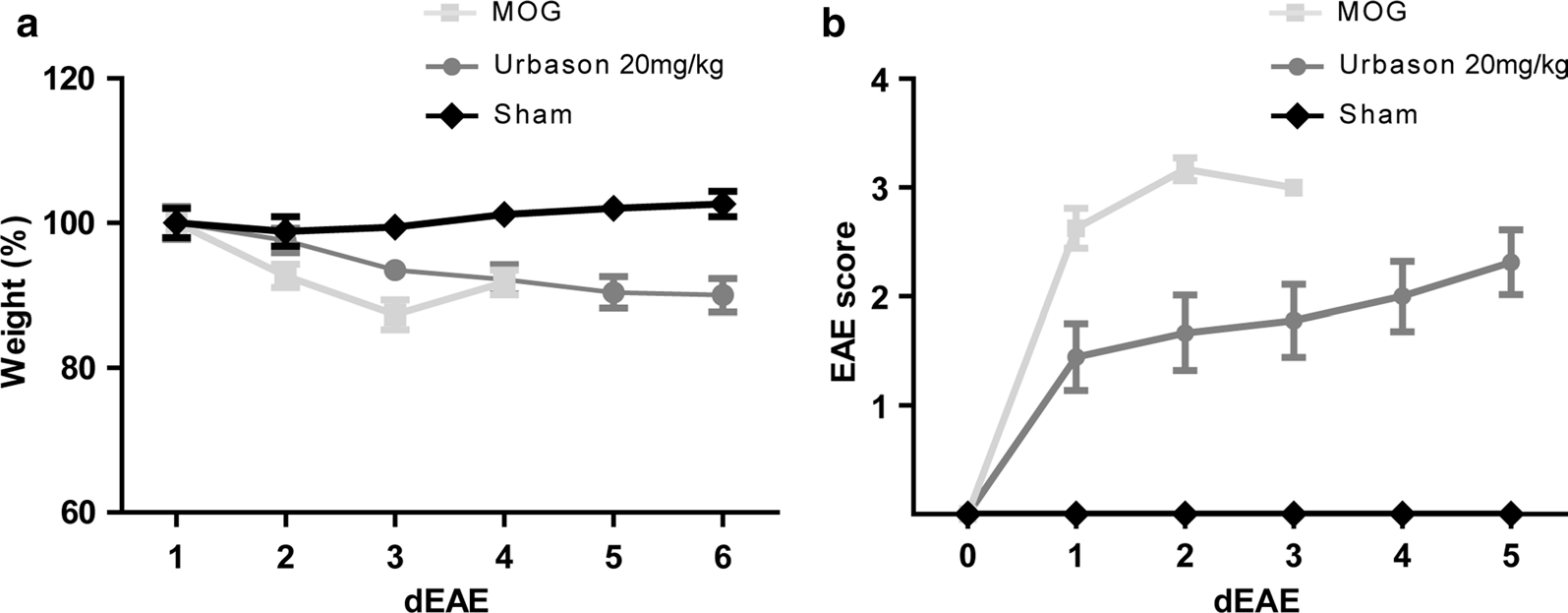
Weight (**a**) and clinical score (**b**) of MOG-immunized Mps- or sham-treated and sham-immunized healthy control animals

To further investigate the usability of fully automated locomotion assessment in therapeutic studies we investigated gait behaviour of MOG-immunized animals under the treatment with Mps, a standardized treatment for MS. The treatment of animals was started at the day of clinical manifestation of EAE and continued for 3 constitutive days with a dose of 20 mg/kg. Animals were clinically followed until day 5 of EAE. The day of disease manifestation did not significantly differ between the groups. Vehicle-treated animals developed symptoms at day 11.67 ± 0.87 (n = 9) post immunization. In the Mps-treated animals first neurological symptoms occurred at day 11.86 ± 1.12 (n = 9) post immunization. Also the severity of symptoms at disease manifestation did not differ significantly in the different groups (vehicle: 2.63 ± 0.52, Mps: 1.44 ± 0.81, P = 0.006) although the tendency to the less severity was observed in Mps-treated animals. In contrast to the control group, movement disturbances were stabilised in the animal group treated with Mps (Fig. 8). Therefore, gait-recording could be successfully performed until day 1 or day 2 of EAE in control and until day 5 of EAE in Mps-treated animals.

Additionally to the classical EAE score, CatWalk analysis was performed in Mps-treated animals and controls. The analysis revealed that all gait parameters (runduration, BOS of hindlimbs or forelimbs, swing speed and stride length) are significantly different between the two experimental groups with better performance in Mps-treated animals (Table 2).

**Table 2:** Results of two-way analysis of variance for each run characteristic in Mps versus control animals

Parameter	Effect	P
Run duration	Group	0.44
	Time	0.31
	Interaction (group × time)	<0.01
Swing speed right front	Group	0.14
	Time	<0.01
	Interaction (group × time)	<0.01
Straight length right front	Group	0.48
	Time	0.19
	Interaction (group × time)	<0.01
Swing speed right back	Group	0.32
	Time	0.22
	Interaction (group × time)	<0.01
Straight length right back	Group	0.14
	Time	0.02
	Interaction (group × time)	<0.01
Swing speed left front	Group	0.21
	Time	<0.01
	Interaction (group × time)	0.89
Straight length left front	Group	0.59
	Time	0.17
	Interaction (group × time)	<0.01
Swing speed left back	Group	0.55
	Time	0.56
	Interaction (group × time)	0.06
Straight length left back	Group	0.62
	Time	0.47
	Interaction (group × time)	<0.01
BOS front paws	Group	0.37
	Time	0.51
	Interaction (group × time)	<0.01
BOS back paws	Group	0.93
	Time	0.68
	Interaction (group × time)	<0.01

## Discussion

CatWalk XT gait analysis system provides the possibility to quantify changes in locomotion of an animal in a highly precise and fully automated way. We analysed a cohort of different dynamic gait parameter in preclinical as well as during the clinical phase of EAE. In this study, we addressed the questions whether early subclinical gait alterations can be detected before overt locomotor dysfunction detected by the standardized EAE score and whether the CatWalk system can be served as a refined behavioral testing for treatment studies. Furthermore, we investigated the timeline for the histopathological changes occurring in the spinal cord in MOG-EAE in BN-rats. The investigation of the different gait parameters did not reveal any abnormalities in the preclinical phase of EAE. Analysing the predictive power of gait parameters in regard to disease onset in this model, we detected that the average of run duration, the average of stride length back right and the average of straight length front right displayed the highest predictive power for the disease onset. The reason for the involvement of the right side is not known. Histopathological analysis revealed that pathological changes in the spinal cord occur simultaneously with the behavioral abnormalities.

Classically the CatWalk was developed to quantify axonal regeneration in rat animal models of traumatic spinal cord injury [11, 13]. In contrast, the application of the behavioral tests in conventional EAE models is limited due to the disseminated and variable nature of the disease makes a correlation of clinical deficits and defined tract system very complex and often impossible. In the MOG-EAE model used in this study the lesions are restricted to the spinal cord and optic nerve renders this model suitable for automated behavioral testing. In the same animal model apoptosis of retinal ganglion cells with decline of visual function in electrophysiological recordings was observed in the preclinical phase of EAE. At this time point no histopathological abnormalities were detected in the optic nerve [12, 14]. In MOG-EAE model in mice axonal loss in the spinal cord was detected before clinical manifestation of the disease [15]. However, it is not clear whether the abnormalities observed by histopathology and electrophysiology leads to the clinical signs detectable in more sophisticated behavioral test. Therefore, in the present study, we aimed to detect gait abnormalities by automated CatWalk system before overt clinical signs in conventional EAE-score. The analysis of the gait parameters did not show any preclinical abnormalities by using automated behaviour test. Similar to our previous study on the optic nerve [12], we found no histopathological abnormalities (inflammatory infiltration and demyelination) in the spinal cord before the manifestation of the disease. In our study, histopathological analysis of the spinal cord showed first signs of inflammation and demyelination at the day of clinical manifestation of EAE. Acute axonal damage was also first present at this time point. These alterations occur with caudal-rostral gradient. In the later disease stages of clinical manifest EAE we detected that the thoracic and the cervical regions of the spinal cord are increasingly affected by the disease. Based on the anatomical organization of innervation of the limbs this histopathological alteration correlates well with partial paresis or weakness of hindlimbs at the disease onset and involvement of the forelimbs during the disease course [16]. Our histopathological data of inflammation, demyelination and axonal damage of the spinal cord are not surprising and are in line with histopathological evidence of close association of inflammation and neurodegeneration at all stages of MS [17].

There are several tentative explanations why we failed to observe preclinical abnormalities by CatWalk system. One obvious possibility would be that the symptoms are not present at these early investigation time points. However, also the limitations of the automated test as well as of the used EAE-model should be taken into account. CatWalk gait system belongs to the category of unforced type of locomotion analysis, in which the animal is free to move at any velocity [18]. Thus, while mimics natural locomotion behaviour more closely this system is not able to ensure the animal to move exactly at the same speed. Also intraindividual and interindividual variability have been reported when using unforced systems in normal rodents [19, 20]. Therefore, speed related alterations could be superimposed and thereby invisible in the statistical analysis. In comparison, in another EAE-model improvement of motor coordination was only apparent in a speed controlled rotarod test and could not be detected by using the traditional scoring system [21]. Experimental model used in our study is relatively heterogeneous in the time point of clinical manifestation of EAE and the severity of symptoms at disease onset. Moreover, moderate to severe deficits occurred during the disease course prevented animals to perform CatWalk test. Furthermore, despite the consistent involvement of the spinal cord in this animal model we found that the pathology is randomly distributed and is not restricted to the specific motor tract system. In our study, disseminated lesions were detected in dorsal, lateral and ventral funiculi contributing to the diversity of symptoms at the disease onset. In contrast, the behavioral testing could be successfully applied in localized EAE model specifically targeting dorsal column of the spinal cord [22].

In aforementioned EAE-studies demonstrating benefit of behavioral test for analysing motor function deficits [21, 22]. EAE course was either monophasic with rapid recovery or relapsing-remitting making the measurements of improvement with behavioral tests possible. In contrast, the disease course of MOG-EAE in BN rats is monophasic with continued worsening of the symptoms. Therefore, to assess whether CatWalk analysis can be used as a test for recovery, animals were treated with Mps, a standard relapse therapy in MS. Assessment of locomotion in diseased MOG-EAE-animals revealed clinical stabilization of the gait parameters after Mps treatment was initiated. This data of manipulation on the immune response with cortisone treatment support hypothesis that inflammation is the principle determinate of reversible neurological disability in an early phase of the disease. However, substantially better performance in CatWalk of animals under Mps treatment was also mirrored in the conventional EAE-scoring paradigm.

## Conclusions

Overall, despite being objective test, the results from CatWalk test obtained during the disease course reflect the far less labor-intensive routinely used EAE score. Our results of no detectable preclinical gait abnormalities and stabilisation of symptoms after initiation of therapy indicate that CatWalk analysis can be used in repair-oriented rather than in neuroprotective treatment studies since neuroprotective treatment should be applied before the manifestation of the disease as have been shown previously in this particular animal model [8, 9, 23]. Being quantitative test CatWalk assessment may provide more sensitive information of locomotion changes in regeneration studies and therefore “unmask” functional improvement not visible in the traditional EAE-scoring paradigm. However, CatWalk analysis should be combined with histopathological and electrophysiological studies to give a more accurate view of structure–function relations of disease processes in EAE.

## Abbreviations

APP: amyloid precursor protein
AUC: area under the curve
BN: Brown Norway
BOS: base of support
CNS: central nervous system
dpi: days post immunization
EAE: experimental autoimmune encephalomyelitis
ED1: CD 68 (macrophages/activated microglia)
EDSS: expanded disability status scale
i.p: intraperitoneal
LAVES: Landesamt für Verbraucherschutz und Lebensmittelsicherheit
LF: left forelimb
LH: left hindlimb
MOG: myelin oligodendrocyte glycoprotein
Mps: methylprednisolone
MS: multiple sclerosis
NaCl: sodium chloride
ON: optic nerve
RF: right forelimb
RH: right hindlimb
ROC: receiver operating characteristic
SC: spinal cord
